# Delineation of colorectal cancer ligand-receptor interactions and their roles in the tumor microenvironment and prognosis

**DOI:** 10.1186/s12967-021-03162-0

**Published:** 2021-12-07

**Authors:** Hexin Lin, Lu Xia, Jiabian Lian, Yinan Chen, Yiyi Zhang, Zhicheng Zhuang, HuaJun Cai, Jun You, Guoxian Guan

**Affiliations:** 1 Department of Colorectal Surgery, The First Affiliated Hospital of Fujian Medical University, 20 Chazhong Road, Fuzhou City, 350001 Fujian China; 2 Xiamen Cell Therapy Research Center, The First Affiliated Hospital of Xiamen University. School of Medicine, Xiamen University, Xiamen, China; 3 Department of Laboratory Medicine, Xiamen Key Laboratory of Genetic Testing, The First Affiliated Hospital of Xiamen University, Xiamen, China; 4 Department of Gastrointestinal Surgery, The First Affiliated Hospital of Xiamen University, Xiamen, China; 5 School of Clinical Medicine, Fujian Medical University, Fuzhou, China

**Keywords:** Colorectal cancer, Ligand-receptor interaction, Immune infiltration, Immunotherapy, Overall survival

## Abstract

**Background:**

Immunotherapies targeting ligand-receptor interactions (LRIs) are advancing rapidly in the treatment of colorectal cancer (CRC), and LRIs also affect many aspects of CRC development. However, the pattern of LRIs in CRC and their effect on tumor microenvironment and clinical value are still unclear.

**Methods:**

We delineated the pattern of LRIs in 55,539 single-cell RNA sequencing (scRNA-seq) samples from 29 patients with CRC and three bulk RNA-seq datasets containing data from 1411 CRC patients. Then the influence of tumor microenvironment, immunotherapy and prognosis of CRC patients were comprehensively investigated.

**Results:**

We calculated the strength of 1893 ligand-receptor pairs between 25 cell types to reconstruct the spatial structure of CRC. We identified tumor subtypes based on LRIs, revealed the relationship between the subtypes and immunotherapy efficacy and explored the ligand-receptor pairs and specific targets affecting the abundance of tumor-infiltrating lymphocytes. Finally, a prognostic model based on ligand-receptor pairs was constructed and validated.

**Conclusion:**

Overall, through the comprehensive and in-depth investigation of the existing ligand-receptor pairs, this study provides new ideas for CRC subtype classification, a new risk screening tool for CRC patients, and potential ligand-receptor pair targets and pathways for CRC therapy.

**Supplementary Information:**

The online version contains supplementary material available at 10.1186/s12967-021-03162-0.

## Introduction

According to the latest estimates of the global cancer burden released by the International Agency for Research on Cancer (IARC) in 2020, colorectal cancer (CRC) is the third most common cancer globally, with the second highest case fatality rate. In recent years, research on advanced CRC has advanced rapidly, and preoperative neoadjuvant therapy and total neoadjuvant therapy have been widely studied in clinical trials. After the publication of the KEYNOTE 177 study in 2020, PD-1 blockade therapy based on the ligand-receptor interaction (LRI) between tumor cells and T cells became the first-line therapy for MSI-H metastatic CRC [1]. Increasing attention has been given to research on intercellular LRIs in CRC treatment.

As an essential component of cell–cell communication, LRIs play a vital role in the development and treatment of cancer. LRIs involve various cells in the tumor microenvironment, and interactions in different cells produce different effects. Current research is mainly limited to the impact of a single ligand-receptor pair between two cell types, but the comprehensive effect of multiple ligand-receptor pairs between different cells is still unclear. With the widespread use of single-cell RNA sequencing (scRNA-seq), several studies have developed algorithms and tools to investigate the effects of LRIs via scRNA-seq data [2, 3], which makes it possible to explore LRIs in a holistic way. However, the entire landscape of LRIs in the colorectal cancer ecological environment has not been fully clarified.

We delineated the existing LRIs between several cell types in the tumor microenvironment by analyzing scRNA-seq data from human CRC samples. After clustering and annotating scRNA-seq data, we calculated the strength of the LRIs between different cells, described the general situation of the LRIs, and reconstructed the spatial structure of CRC by LRI strength, which will allow us to explore the pattern of cell–cell communication. Furthermore, based on the strength of the LRIs, we identified malignant epithelial cell subtypes and CRC subtypes and revealed differences in the tumor microenvironment and changes in immunotherapy-related factors. In addition, a survival model and ligand-receptor network were established to explore the impact of ligand-receptor interactions on the survival of CRC patients.

## Material and methods

### RNA-seq data acquisition

The scRNA-seq data of 29 human CRC samples containing 55,543 cells was obtained from the Gene Expression Omnibus database (http://www.ncbi.nlm.nih.gov/geo/) from two studies, and the accession numbers were GSE132465 and GSE144735 [3]. Three bulk RNA-seq profiles of CRC samples were accessed from the TCGA database (https://portal.gdc.cancer.gov/) and the Gene Expression Omnibus database (Accession Numbers GSE39582 and GSE17538). The RNA-seq data of CRC cell lines was downloaded from CCLE (https://sites.broadinstitute.org/ccle).

### scRNA-seq data processing

The ‘Seurat’ package was used to analyze the matrix of unique molecular identifier (UMI) counts per gene. Cell selection was based on the following criteria: cells with > 1000 UMI counts, > 200 genes, < 6000 genes and < 5% mitochondrial gene expression in UMI counts. The ‘scTransform’ package and ‘harmony’ package were used for batch correction and scRNA-seq data integration. The t-distributed stochastic neighbor embedding (tSNE) algorithm was used for dimensionality reduction and cluster classification analysis. The number of principal components (NPCs) was selected based on the elbow plot, and the resolution was determined by the number of cells. Adjusted P-value < 0.05 and log2(fold change) > 0.5 were considered the cutoff thresholds for identifying marker genes. Clusters were annotated by the ‘singleR’ package, marker genes and annotation file of the original research. The ‘Monocle’ package was utilized to construct single-cell pseudotime trajectories of dendritic cells (DCs).

### Ligand-receptor pair interaction strength calculation and analysis

The 1893 currently confirmed ligand-receptor pairs were downloaded from the published database and research [4]. The strength of the LRIs was calculated by referencing the research of Lei Zhang et al. [5]. The LRI strength calculation is briefly described as follows. For single-cell samples, the average ligand expression value of cell type A is multiplied by the average receptor expression value of cell type B. For bulk samples, the ligand expression value of the same sample is multiplied by the receptor expression value. The statistical significance of the ligand-receptor pairs between different pairwise cells was achieved by 1000 permutation tests. The reconstruction of intercellular spatial structure in the scRNA-seq data was performed by Cellular Spatial Organization mapper (CSOmap) based on the LRIs [6]. Cells that were located away from the LRI were defined as those that are 40 units away from the origin. Differentially expressed pairs (DEPs) were screened by the 'edgeR' package, and significantly DEPs were defined as having adjusted P-value < 0.05 and log2(fold change) > 0.5. Database for Annotation, Visualization and Integrated Discovery (DAVID, https://david.ncifcrf.gov/home.jsp) v6.8 was used to perform functional enrichment analyses of the differentially expressed genes, and significantly enriched terms were defined as having Benjamini-corrected P-value < 0.05. Whole genome expression profile data were analyzed by gene set enrichment analysis (GSEA 4.1.0).

### Ligand-receptor subtypes classification of CRC

An unsupervised k-means subclustering algorithm was applied to classify LRI subtypes in the three bulk RNA-seq datasets through ligand-receptor expression patterns. The ‘ConsensusClusterPlus’ package was used to perform unsupervised consensus clustering. The clustering procedure was iterated 1000 times, with 80% sampling in each iteration. The optimal number of clusters was determined by CDF curves and a consensus heatmap. The 50 most significantly upregulated ligand-receptor pairs were selected by the ‘edgeR’ package as the characteristic ligand-receptor pairs of each subtype. A silhouette value was used to evaluate the clustering effect, and the closer the silhouette value was to 1, the better the clustering effect was. The unsupervised subclass mapping method (SubMap; https://cloud.genepattern.org/gp/) was applied to evaluate the similarity between the different subtypes in the three bulk RNA-seq datasets [7]. A Bonferroni-corrected P-value < 0.05 was considered statistically significant. Then, Kaplan–Meier (KM) survival curves were used to evaluate the overall survival (OS) of patients with different LRI subtypes. The log-rank test was used to analyze the significance of survival differences between groups. The characteristic ligand-receptor pairs of each subtype were used to identify the LRI subtype of the single-cell samples. The abundance of tumor-infiltrating lymphocytes (TILs) and efficacy of immune checkpoint blockade therapy in the bulk RNA-seq samples were estimated by ImmuCellAI (http://bioinfo.life.hust.edu.cn/ImmuCellAI#!/), a tool used to estimate the abundance of immune cells and predict the response to immunotherapy from a gene expression dataset [8]. The correlation between the LRI intensity and the abundance of TILs was analyzed using Pearson correlation. Submap was utilized to analyze the consistency of the expression profiles of immunotherapy response and the LRI subtypes.

The “maftools” R package was utilized to analyze the single nucleotide polymorphism (SNPs) and copy number variations (CNVs) of ligand-receptor interaction subtypes in TCGA-COREAD cohort. The mutation information of CRC cell lines was downloaded from CCLE. The GISTIC 2.0 was applied to define the amplified and deleted regions of each subtype [9]. The G-score is calculated by weighing regions of aberration against the likelihood for random occurrence through permutation test [10].

### Co-culture and reactivity assay

Peripheral blood was collected from healthy volunteer from the First Affiliated Hospital of Fujian Medical University. Peripheral blood mononuclear cells (PBMC) were isolated with Ficoll–Hypaque by density gradient centrifugation. Cell culture dish were coated with 10 μg/mL anti-CD3 (novoprotein, GMP-A018) and anti-CD28 (GMP-A063) and kept overnight at 4 °C. PBMC were culture in RPMI 1640 with 10% fetal bovine serum, 1:100 Penicillin–Streptomycin and 500 U/ml IL-2 (novoprotein, c013). Half of the medium, including IL-2, was refreshed every other day. To evaluate the activation of CD8 + T-cells, cells were washed with PBS and stained with human anti-CD3-APC(Biolegend), anti-CD4-FITC(Biolegend), anti-CD8-PE(Biolegend) for 30 min at 4 °C. Cells were washed twice with PBS and recording at flow cytometer (BD FACSCanto II).

CRC cell lines (HCT116, CACO2, SW480, HT29) were seeding in 96-well plates with 5000 cells/well for 24 h. The next day, CRC cells were pretreated with signaling pathways inhibitors, including PI3K/AKT inhibitors (LY294002, PI-103), MEK/ERK inhibitors (PD98059, FR 180204), TNF-α inhibitor (R-7050, Geraniin) and TGF-β inhibitor (A83-01, SB-431542). All pathway inhibitors were purchased from MCE. The effect of inhibitors on CRC cell was detected by CCK8 kit (Meilunbio).

Activated CD8 + T-cells were seeded at a 10:1 effector:target ratio in 96-well plates. Co-culture was performed in T cell medium for 24 h. Lactate dehydrogenase (LDH) kit (Beyotime) was used to measure the cytotoxicity of CD8 + T cells to CRC cells.

### Generation and validation of the LRI prognostic risk score model

Univariate analysis was performed to determine the survival-related ligand-receptor pairs in the GSE39582 dataset. The survival-related pairs with P-values < 0.05 in univariate analysis were further filtered by least absolute shrinkage and selection operator (LASSO) regression analysis. Multivariate Cox regression was used to calculate the regression coefficients and the prognostic risk score. The CRC patients in the bulk RNA-seq datasets were classified into either a low-risk (low score) group or a high-risk (high score) group according to the median value. Thirty percent of the GSE39582 dataset was used as the internal validation group, and the TCGA colorectal adenocarcinoma and rectum adenocarcinoma (TCGA-COREAD) and GSE17538 datasets were used as the external validation group to jointly verify the survival prediction ability of the ligand-receptor pair risk score. The risk score combined with clinicopathological factors was used to construct an LRI prognostic model. The predictive accuracy of the prognostic model was assessed by time-dependent receiver operating characteristic (ROC) curve analysis within 3 years and 5 years. A nomogram was drawn using the ‘rms’ package. Calibration plots for 3-year and 5-year OS predictions were constructed to assess the calibration and discrimination of the survival model.

## Results

### Discovery of LRI patterns in human CRC using scRNA-seq data

**Fig. 1:**
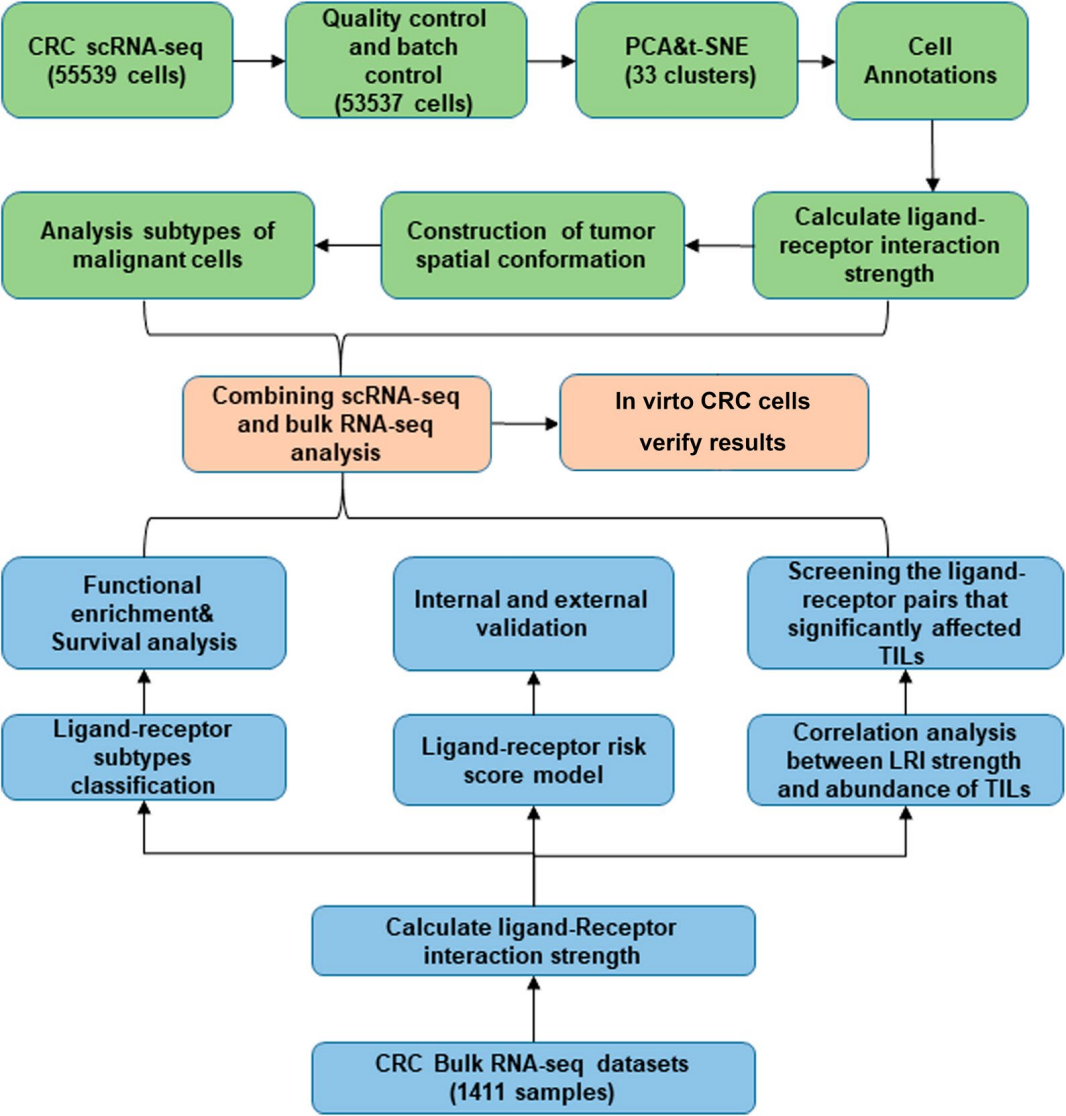
Flowchart of the study design and process

**Fig. 2:**
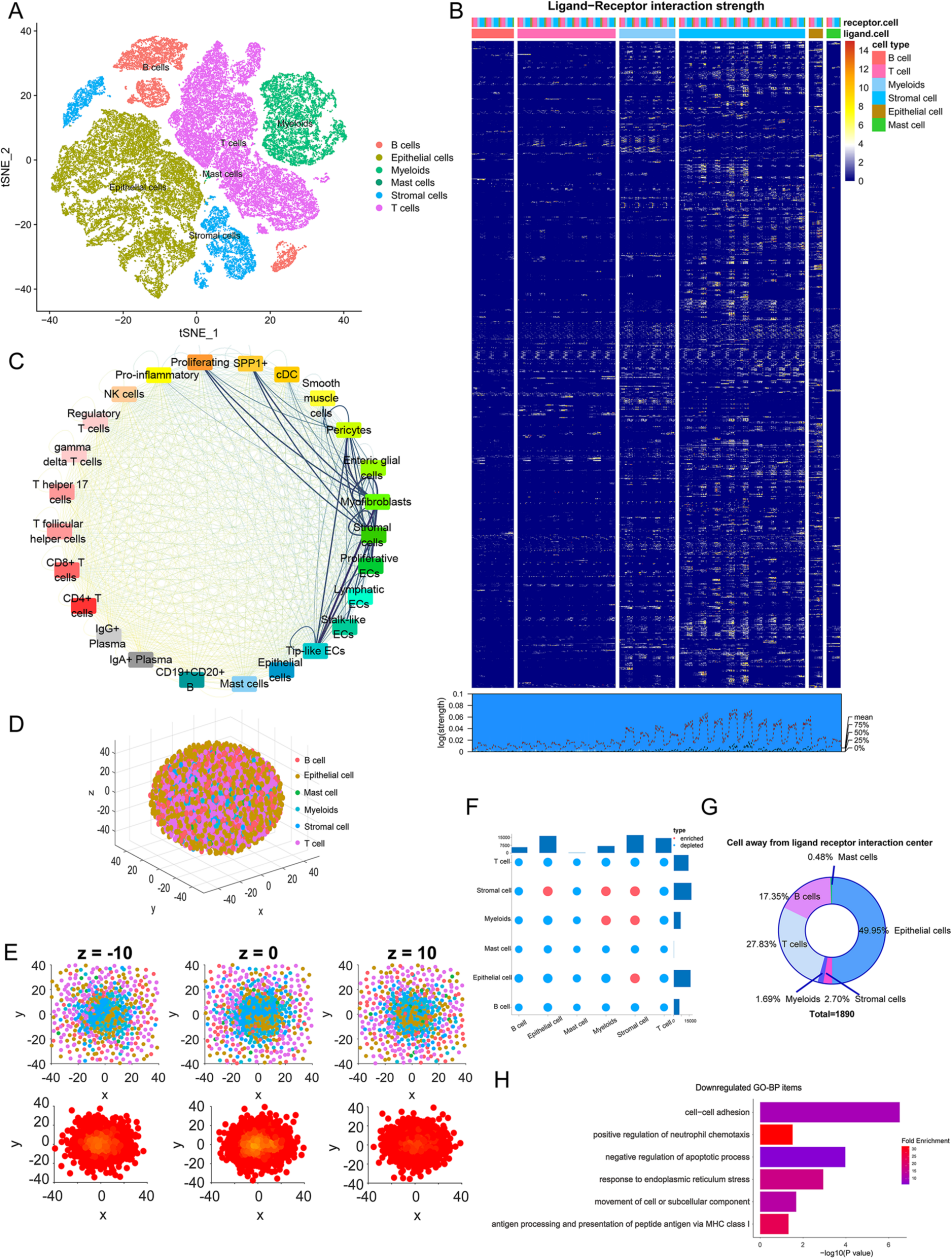
Ligand-receptor pair interaction strength calculation and analysis. **A** t-SNE plot showing clusters of 6 main cell types from 25 CRC scRNA-seq samples. **B** Description of the strength of 1733 ligand-receptor pairs’ interactions strength. The heatmap shows ligand-receptor pairs’ interaction strength between 6 main classes and 25 subclasses cells. The density plot shows average interaction strength between the different cell types. **C** Network plot diagram shows ligand-receptor pair interactions in 24 cell types. **D** CSOmap was used to reconstruct the three-dimensional spatial structure of the 25 CRC scRNA-seq samples. **E** Diagrams and density plot of different longitudinal sections of tumor tissue spatial structure. **F** The statistical significance of interactions between different cell types. **G** Proportion of cell types away from the interaction center. **H** Functional enrichment of the downregulated DEGs in malignant epithelial cells far from the interaction center

A schematic diagram of our research process is presented in Fig. 1. After batch calibration and quality control of two CRC scRNA-seq datasets, 53,537 CRC cells were further studied. The tSNE algorithm was applied to classify all cells into 33 clusters, and six main types and 25 kinds of cells were annotated by ‘singleR’ and the original annotation files (Fig. 2A, Additional file 4: Fig. S1). Then, we calculated the strength of the LRIs from these cells. The heatmap and density plot of CRC tissue cells illustrated that the strength of ligand-receptor pairs was high between myeloid cells and stromal cells (Fig. 2B). Through the circle plot of LRIs in CRC, we found that myeloid cells and stromal cells are at the center of the interaction pattern, especially endothelial cells, fibroblasts, and stromal cells (Fig. 2C). Then, we used CSOmap to reconstruct the spatial relationships in CRC tissue. We found that the interaction between myeloid and stromal cells and malignant epithelial cells constitutes the center of the tumor, and T cells and B cells do not interact closely with other cells (Fig. 2D–F). In addition, malignant epithelial cells have substantial heterogeneity. Some of the malignant epithelial cells were clustered in the spatial center of the tumor, while some were separated from the interaction center. Analysis of the DEGs in these two types of malignant epithelial cells revealed that epithelial cells far from the LRI center showed downregulation in certain terms, including cell adhesion, negative regulation of apoptotic process, antigen processing and presentation via MHC class I and positive regulation of neutrophil chemotaxis (Fig. 2G, H, Additional file 1).

### Identification of LRI subtypes in colorectal cancer

**Fig. 3:**
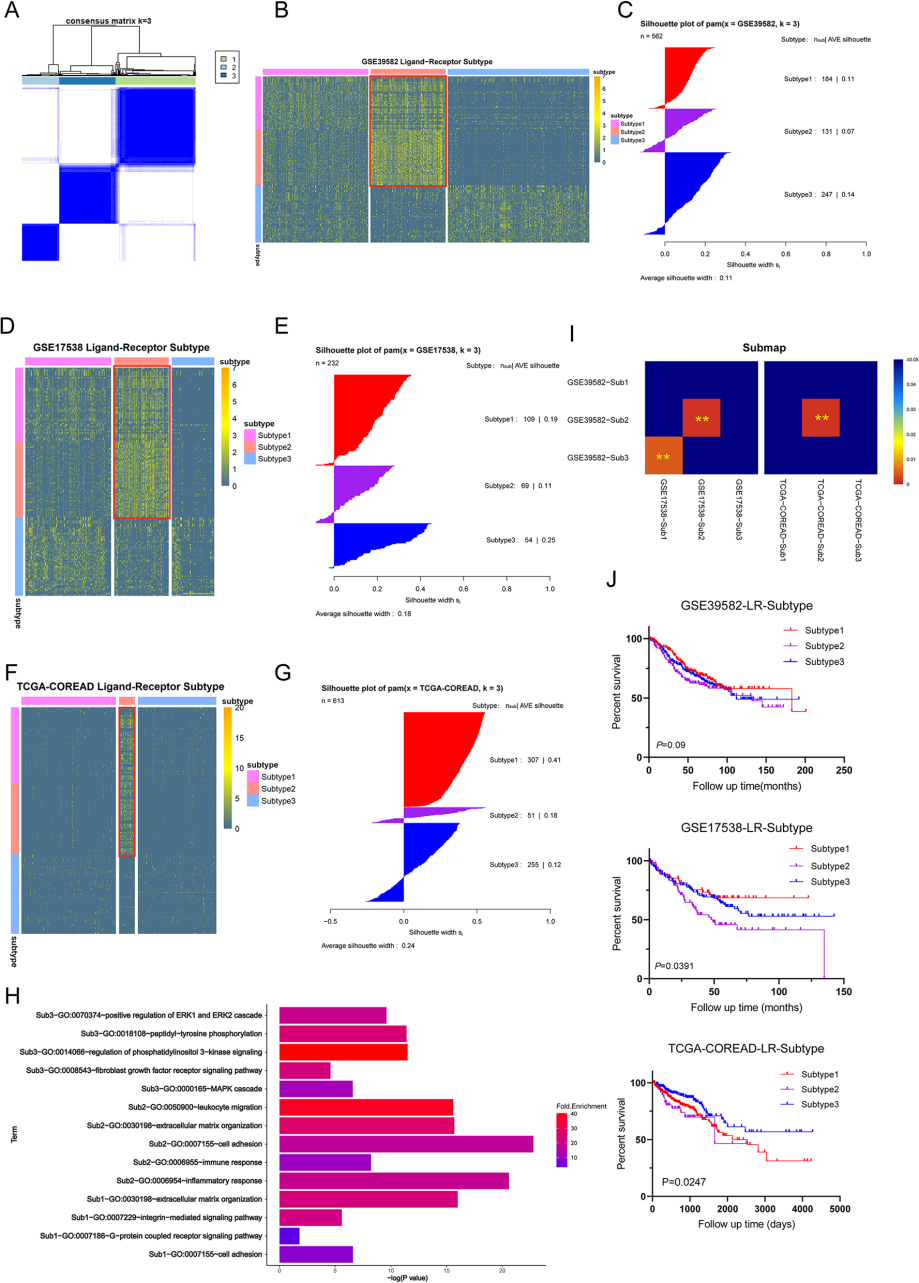
Identification of ligand-receptor pair interaction subtypes in CRC bulk RNA-seq data. **A** Consensus clustering matrix for the optimal cluster number (k = 3) in the GSE39582 dataset. **B**, **D**, **F** Heat maps of marker genes for three ligand-receptor pair subtypes. **C**, **E**, **G** The consistency between each sample and each subtype was evaluated by the silhouette value. **H** GO-BP enrichment analysis results of the marker genes of each subtype. **I** The consistency of the identified subtypes in the three bulk RNA-seq datasets can be found through submap analysis. **J** Kaplan–Meier survival analysis was used to compare the survival differences of each receptor subtype. **P < 0.01

To distinguish the LRI subtypes of patients with CRC, we calculated the LRI strength in the three bulk RNA-seq datasets (GSE39582, GSE144735, and TCGA-COREAD). An unsupervised analysis was performed on CRC patients from the GSE39582 dataset using consensus clustering. According to the consensus heatmap, the optimal number of clusters was determined to be three (Fig. 3A). The top 50 upregulated marker pairs of each subtype in GSE39582 were identified, and subtype2 could be well distinguished in the three datasets (Fig. 3B–G, Additional file 2). Each subtype in the different datasets was mapped through a submap, and the results showed that the LRI patterns of subtype2 in the three datasets were significantly matched (Fig. 3I). Functional enrichment analysis of each subtype’s marker pairs was performed. We found that the GO-BP terms mainly enriched in subtype1 included cell adhesion, integrin receptor and G protein-coupled receptor pathway. Subtype2 was significantly enriched in immune-, inflammation- and cell adhesion-related terms. Subtype3 was enriched in terms related to the fibroblast growth factor receptor pathway, MAPK pathway and ERK cascade (Fig. 3H). In addition, we found that the prognosis of patients with subtype2 was worse than that of patients with the other two subtypes (Fig. 3J). We found that the expression of immunotherapy-related genes (PDL1, PDL2, CTLA4, CD80, and CD86) were significantly higher in subtype2 than in the other two subtypes (Additional file 5: Fig. S2A–C). Furthermore, the expression pattern of subtype2 patients matched that of patients with a poor response to immune checkpoint blockade therapy (Additional file 5: Fig. S2D).

We further analyzed the differences of genomic alterations between the ligand-receptor subtypes in the TCGA-COREAD dataset. We separately showed the significantly mutated genes among the three subtypes (Additional file 6: Fig. S3A). In addition, we determined the mutations associated with targeted therapy for CRC among subtypes (Additional file 6: Fig. S3B). The mutation of oncogenic signaling pathways were analyzed, and it was found that the mutant genes in subtype2 were less than those in the other two subtypes (Additional file 6: Fig. S3C). Tumor mutation burden (TMB) is a biomarker to predict clinical response to immunotherapy, it was found that the subtype3 had the lowest TMB, and significantly lower than subtype1 (Additional file 6: Fig. S3D). The fraction of genome altered of subtype2 had lower levels (Additional file 6: Fig. S3E). We further delineate the significantly CNVs of each subtype (Additional file 6: Fig. S3F). In each subtype, functional enrichment of genes which the expression levels were affected by CNV was applied. We found that subtype1 was enriched in cytokine receptor binding and subtype2 was enriched in phospholipid binding and oxidoreductase activity, and subtype3 has no specific enrichment function.

### Analysis of ligand-receptor subtype at the scRNA-seq and cell level

**Fig. 4:**
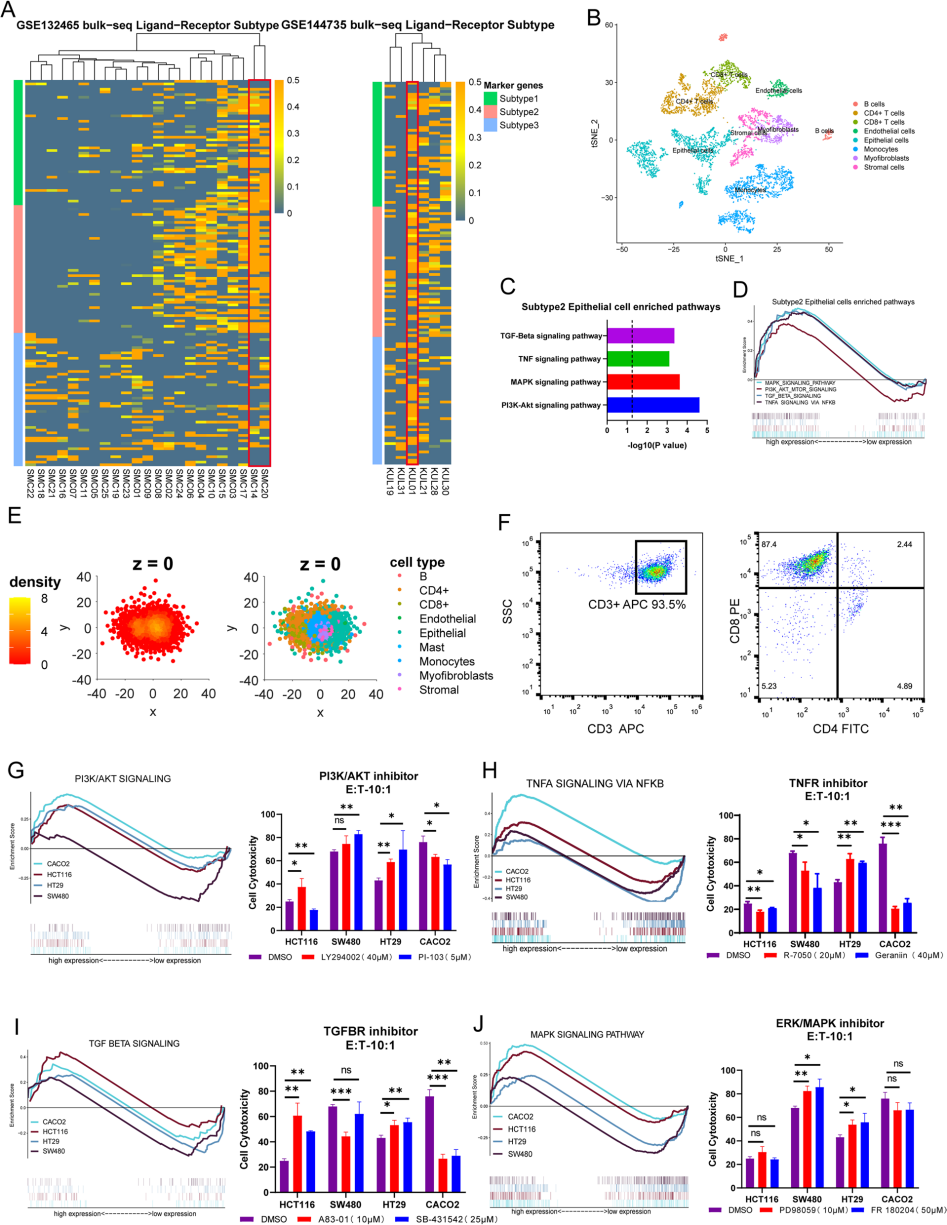
Analysis and verification of ligand-receptor subtype 2 in CRC cell lines and scRNA-seq data. **A** scRNA-seq data of 29 CRC single-cell samples were clustered according to the subtype marker ligand-receptor pairs. **B** t-SNE plot of the scRNA-seq data of SMC14, SMC20 and KUL01. **C** Pathway enrichment analysis of DEGs between subtype 2 and other subtypes of malignant epithelial cells. **D** GSEA analysis of differential pathways of malignant epithelial cells in scRNA-seq sample. **E** 3D visualization of the spatial structure of SMC14, SMC20 and KUL01 (right panel). Cell types and density scatter diagrams of longitudinal sections of tumor tissue spatial structure (left panel). **F** Flow cytometry analysis results of activated CD8 + T-cells. **G**–**J** left panel: GSEA analysis of PI3K/AKT, TNFA, TGF-Beta and MAPK pathways in 4 CRC cell lines. **G**–**J** right panel: LDH level measured the cytotoxicity of CD8 + T-cell to co-cultured tumor cells in vitro. E:T, effector:target ratio. *P < 0.05, **P < 0.01, ***P < 0.001

As mentioned earlier, we identified a ligand-receptor subtype that showed upregulation in immune-inflammation-related pairs in the bulk RNA-seq datasets. Next, cluster analysis was performed on two scRNA-seq datasets, and the results showed that the LRI patterns of KUL-01, SMC-14 and SMC-20 were consistent with the characteristics of subtype2 (Fig. 4A). The cell interactions of subtype2 CRC were explored by spatial reconstruction (Fig. 4C). We obtained a significant difference from the previous tumor model of all specimens, where T cells were more closely linked to themselves and other cells, and the density plot illustrated the appearance of another interaction center (Fig. 4D). In addition, we found that tumor cells of subtype2 was significantly enriched in PI3K-AKT, MAPK, TNF, and TGFB pathways, (Fig. 4), and the effect of inhibitors of these pathways on CRC cells vitality was verified by CCK8 experiments (Additional file 7: Fig. S4A).

In order to verify whether subtype2 affects the interaction with T cells through the above-mentioned pathways, and further affects the survival of CRC patients and the efficacy of immunotherapy, we conducted the co-culture of tumor cells and CD8 + T cells. We isolated T cells from the peripheral blood of healthy people and activated the main tumor-killing component, CD8 + T-cells (Fig. 4F). The activated CD8 + T-cells were co-cultured with tumor cells treated with different pathway inhibitors. The results of LDH assay showed that PI3K inhibitor and MAPK inhibitor could significant promoted the tumor-killing ability of CD8 + T-cell in SW480 and HT29 (Fig. 4G, J right side). After TNF receptor inhibitor treatment, LDH was significantly reduced and the tumor killing ability was weakened in HCT116, SW480 and CACO2 (Fig. 4H right side). After TGF-β receptor inhibitor treatment, the tumor killing ability was weakened in SW480 and CACO2, HCT116 and HT29 had the opposite phenotype (Fig. 4I right side). By analyzing the transcription profile and mutation profile of these cell lines, it is found that significant differences exist in genetic background (Fig. 4G–J left side, Additional file 7: Fig. S4B).

### Effect of LRI on tumor immune infiltration in CRC

**Fig. 5:**
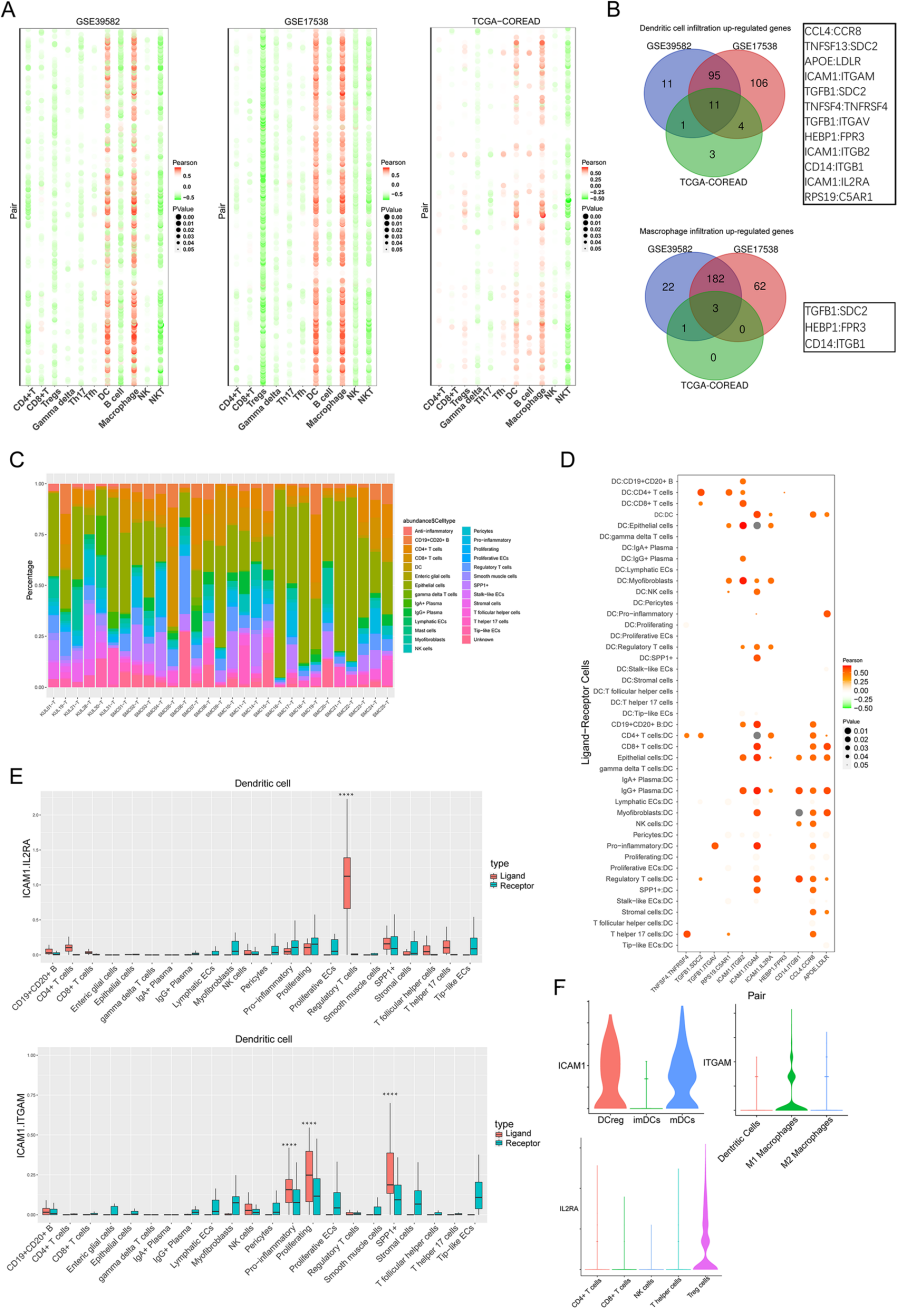
Ligand-receptor interactions affect tumor-infiltrating immune cells. **A** Pearson’s correlation analysis was performed between the ligand-receptor interaction strength and the abundance of tumor-infiltrating immune cells in the bulk RNA-seq data. **B** The ligand-receptor pairs that collectively influence DC and macrophage infiltrates in three datasets. **C** The proportion of tumor-infiltrating cells in 29 single-cell samples. **D** Correlation analysis of the 9 ligand-receptor pairs’ interaction strength and abundance of DCs in the scRNA-seq data. **E** The interaction strength and significance of two LR pairs (ICAM1:IL2RA and ICAM1:ITGAM) between DCs and other cell types. The significance was calculated by permutation tests. **F** Expression of ICAM1, IL2RA, and ITGAM in the CRC scRNA-seq data. Th17, T helper 17 cells; Tfh, T follicular helper cells; DC, dendritic cells; ECs, endothelial cells; DCregs, regulatory dendritic cells; mDCs, mature dendritic cells; imDCs, immature dendritic cells. ****P < 0.0001

To investigate the relationship between TILs and LRIs, we analyzed the correlation between LRI intensity and the abundance of B cells, T cells, DCs, macrophages, and NK cells in three datasets (Fig. 5A). The results demonstrated that LRIs significantly influenced the infiltration of DCs and macrophages in all three datasets. The intersecting ligand-receptor pairs of the three datasets showed that TGFB1:SDC2, HEBP1:FPR3, and CD14:ITGB1 were significantly positively correlated with the abundance of DCs and macrophage infiltration (Fig. 5B). The other 9 ligand-receptor pairs were significantly associated with DC infiltration. We further explored the role of these ligand-receptor pairs on DCs using scRNA-seq data. We assessed the infiltration of various types of cells in the sample (Fig. 5C). We analyzed the correlation between the strength of the LRI and the proportion of DC infiltrates, as well as the significance of the difference in the strength of ligand-receptor pair interactions between DCs and other cells (Fig. 5D, E, Additional file 8: Fig. S5). Through correlation analysis and strength analysis of the LRIs, we found that through ICAM1:IL2RA, the specific interaction among mDCs, DCregs and regulatory T cells affects the infiltration of DCs in CRC tissues. In addition, ICAM1 expressed on mDCs and Dregs interacts with ITGAM on M1 macrophages, significantly and specifically affecting the infiltration of DCs. The clustering process for monocytes and DCs is shown in Additional file 9: Fig. S6.

### Generation and validation of the LRI-based prognostic risk score model to predict CRC patient survival

**Fig. 6:**
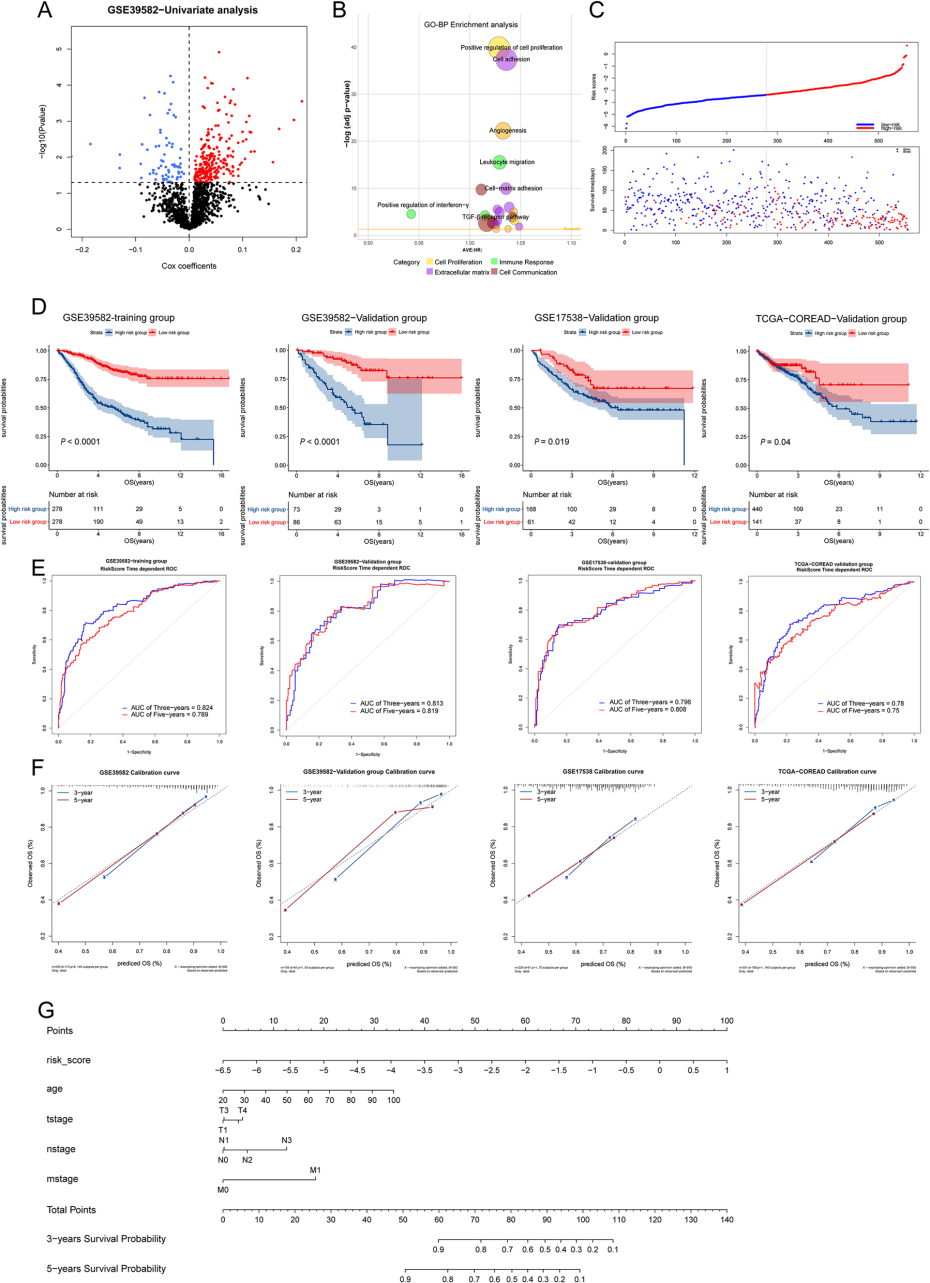
Construction of the prognostic model of CRC patients based on ligand-receptor pairs. **A** Volcano plot showing ligand-receptor pairs that significantly influence survival in the GSE39582 dataset. **B** The bubble plot shows the mean hazard ratios (HRs) of all genes in each GO-BP term enriched in survival-related ligand-receptor pairs. **C** Risk curve (upper panel) and dot plot (bottom panel) describe the relationship between the risk score and patient survival status. **D** KM survival analysis illustrated the survival condition of high-risk and low-risk patients in the training group, internal validation group and external validation group. **E** ROC curve analysis was performed to estimate the prognostic capacity of the ligand-receptor pair predictive model. **F** Calibration plots of the prognostic model for predicting survival rates in all cohorts. **G** The nomogram of the CRC prognostic model was constructed based on the ligand-receptor risk score and clinicopathological data in GSE39582

**Fig. 7:**
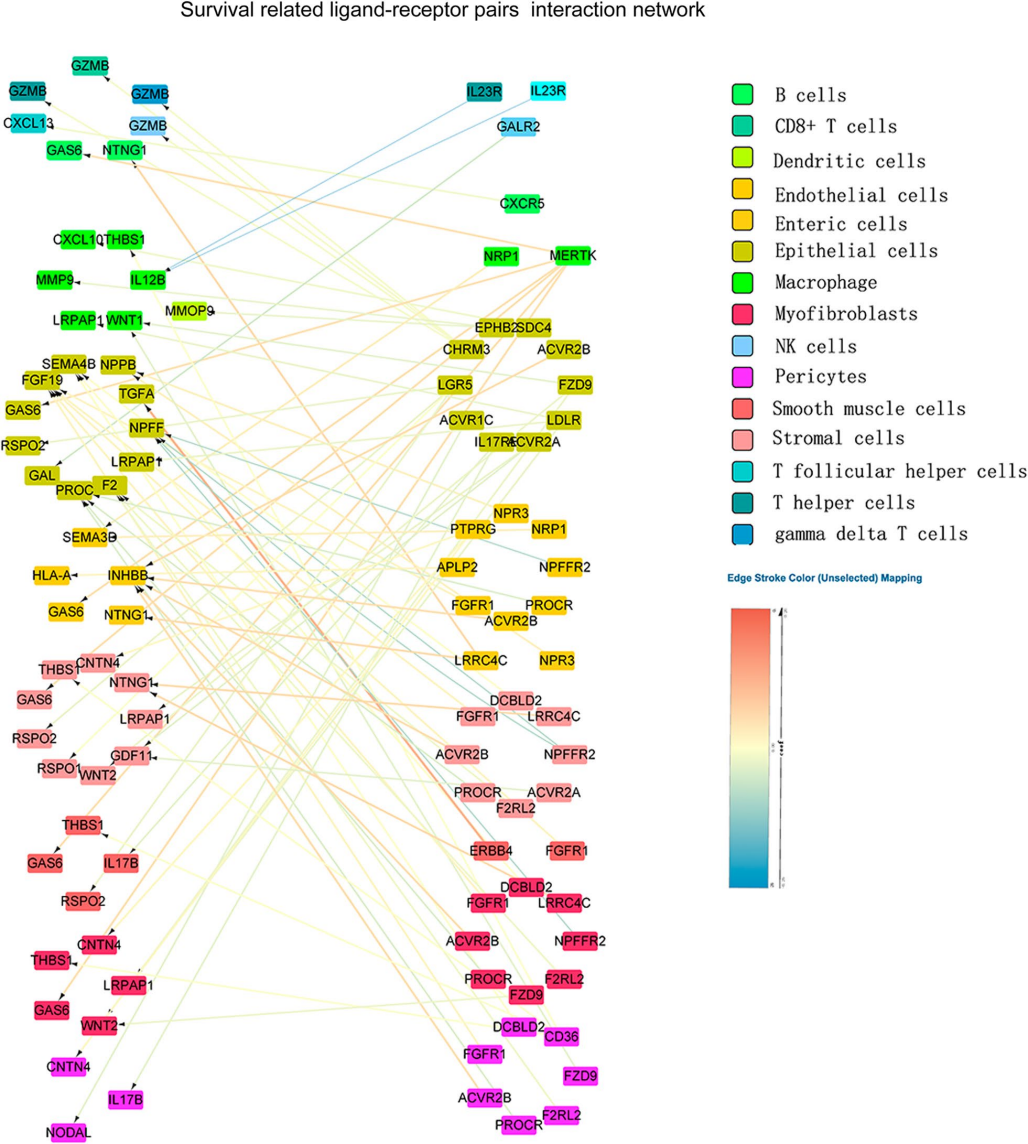
CRC survival-related ligand receptor interaction network. The color of the line between the ligand receptors represents the regression coefficient

As mentioned earlier, the different LRI subtypes are related to different prognostic outcomes. To better explore the influence of LRIs on the survival of CRC patients, we constructed a prognostic prediction model for CRC patients. Univariate Cox analysis was performed and identified 421 prognosis-associated ligand-receptor pairs in the GSE39582 training set (Fig. 6A, Additional file 3). Bubble plots showed the ligand-receptor pairs that influence prognosis in CRC patients, and these pairs were mainly involved in cell proliferation, extracellular matrix, cell communication, and immune response (Fig. 6B). LASSO regression followed by multivariate Cox analysis was performed, and 30 survival-related pairs were identified (Additional file 10: Fig. S7A–C). 30 ligand pairs were further analyzed by functional enrichment analysis (Additional file 11: Fig. S8). The prognostic risk score was developed based on these pairs. Risk scores were calculated for all patients in the training set (GSE39582) and validation set (GSE144735 and TCGA-COREAD), and the patients were divided into either a high-risk (high score) group or a low-risk (low score) group using the median value of the risk score as the cutoff value (Fig. 6C). KM survival analysis demonstrated that patients in the high-risk group had significantly poorer OS than those in the low-risk group (Fig. 6D). We constructed a prognostic model including the risk score combined with clinicopathological data and drew a nomogram (Fig. 6G). Time-dependent ROC analysis indicated that this model showed excellent performance in predicting the 3-year and 5-year survival rates of CRC patients (Fig. 6E). The calibration plots showed that the predictions were in concordance with the actual observations for the 3-year and 5-year survival rates in the training set and validation set (Fig. 6F). The survival-related LRI network was delineated to further demonstrate the intercellular interactions of these pairs (Fig. 7). These findings suggested the appreciable reliability of the prognostic model, which can be applied in diverse CRC patients.

## Discussion

In the tumor microenvironment, the communication between different cell types is associated with mechanisms of oncogenesis, tumor progression, therapeutic resistance, immune infiltration, and inflammation [2, 11]. scRNA-seq is an effective method to analyze the LRIs that occur in CRC [3, 5]. With the development of cell–cell interaction-based immunotherapy in colorectal cancer, it is crucial to understand the existing ligand-receptor pairs further. By analyzing scRNA-seq data from human and mouse CRC, Lei et al. also revealed that DCs and macrophages are key regulators of cell communication in the tumor microenvironment and further identified the specific target of anti-CSF1R treatment [12]. Our study of the existing ligand-receptor pairs can provide a holistic and realistic view of cell-to-cell communication in the tumor microenvironment. By analyzing LRI strength, we found that myeloid cells and stromal cells were the core of LRIs.

As early as 2011, Piero Dalerba carried out single-cell research, exploring the multilineage differentiation processes and cellular heterogeneity of CRC [13]. Intratumoral heterogeneity plays an important role in the resistance to cancer therapy, and finding new intratumoral cell types can help to identify therapeutic targets and explore drug resistance [14]. We identified a subtype (subtype2) of malignant epithelial cells characterized by low cell adhesion, inhibition of neutrophil chemotaxis, and inhibition of antigen presentation, which has the potential for immune escape and distant metastasis. We further analyzed the differences between subtypes at the SNP and CNV levels, and found that subtype2 was characterized by lower oncogenic-related mutations and lower fraction genome altered. The spatial reconstruction of tumors based on LRIs, which was accomplished by using CSOmap, a computational tool for inferring cell-to-cell interactions, makes up for the lack of spatial information in single-cell data and provides validation for the ligand-receptor subtypes. In addition, we identified an LRI subtype in the transcriptome of CRC patients that shows upregulation in chemokines and intercellular adhesion-related ligand-receptor pairs. Chemokines play an important role in immune and inflammatory responses, are involved in CRC progression and metastasis and are also associated with poor prognosis, which is consistent with our findings [15, 16]. In addition, analyzing the related pathways of tumor cells in the subtypes shows that pathways related to immune escape are significantly enriched, including PI3K/AKT, TGF-β, and MAPK pathways. Previous studies have suggested that inhibiting these pathways can reverse tumor immune escape [17–19]. Our research results also found that these pathways inhibitors can enhance the cytotoxicity of CD8 + T-cells when co-cultured with some CRC cell lines. However, the role of TNF in tumors is a double-edged sword. On the one hand, it kills tumor cells through cytotoxicity, and on the other hand, it also participates in the occurrence and development of tumors. TNFR inhibitors can significantly decreased anti-tumor activity of CD8 + T-cells co-culture in vitro experiment. However, co-culture systems may have different responses to inhibitors due to differences in genetic background between different cell lines and the effect of inhibitors on tumor cell viability.

Through the analysis of the influencing factors of TILs, we revealed that the infiltration of monocytes in CRC tissues was significantly affected by LRIs. We also found that ICAM1:IL2RA and ICAM1:ITGAM could increase the infiltration of DCs by interacting with Tregs and monocytes/macrophages, respectively. ICAM-1 plays an important role in leukocyte-mediated inflammation and T cell activation and was found to interact with ITGAM as early as 1990 [20]. Previous studies have shown that ITGAM-encoded CD11b (MAC-1) is highly expressed on macrophages and mediates intercellular adhesion [21], which was also observed in the CRC scRNA-seq datasets. Few studies on the function of ICAM1:ITGAM [22] and no studies on this pair between DCs and macrophages have been found before. We found that the infiltration of mDCs in tumor tissues can be promoted by the expression of ICAM1 and interaction with ITGAM expressed in macrophages.

Furthermore, several studies have indicated that Tregs can downregulate the maturation process of DCs and abrogate the antigen-presenting capacity of DCs [23–25]. Previous studies have shown that IL2 is crucial for Treg development and maturation, and the long-term survival of Tregs requires continuous IL2 signaling [26]. Here, we speculate that ICAM1 inhibits IL2RA through competitive binding with IL-2 and inhibits the inhibitory effect of Tregs on DCs.

In previous research, prognostic risk models of CRC based on LRI have not been constructed; however, many prognostic models have included ligand-receptor pair-related genes [27–30], and several genes in this model have been shown to influence CRC development [31–36]. Our survival model based on LRIs has good predictive efficacy for 3-year and 5-year survival rates, providing a new tool for the early assessment of adjuvant intervention in patients with CRC.

The current study has some limitations. The single-cell data lacked spatial information, and we used spatial reconstruction, but further spatial transcriptome verification is needed. The factors affecting TILs in this study need to be further verified by in-depth experiments. This prognostic model has yet to be further validated for clinical effectiveness in a larger cohort of CRC patients.

## Conclusions

We delineated the LRI network of CRC using scRNA-seq and bulk RNA-seq datasets and calculated each ligand-receptor pair’s strength across different cells. We identified a malignant epithelial cell subtype with potential for distant metastasis and immune escape and a CRC subtype that showed upregulation in immunoinflammatory ligand-receptor pairs, which is also associated with CRC immunotherapy response and survival. And it was found that this CRC subtype induces the hypo-responsiveness of CD8 + T-cells by regulating PI3K/AKT, MAPK, TGF-Beta and TNFA pathways. We explored ligand-receptor pairs affecting TILs and found that ICAM1:IL2RA and ICAM1:ITGAM specifically affected the infiltration of DCs. We also constructed a prognostic prediction model for colorectal cancer based on LRIs to predict patients' prognostic risk and provide suggestions for further guiding treatment strategies.

## Supplementary Information

**Additional file 1. **DEGs between EPI cells far from the interaction center and EPI cells from the interaction center.

**Additional file 2.** Subtype1-Marker ligand-receptor pairs.

**Additional file 3.** Univariate Cox analysis prognosis-associated LR pairs.

**Additional file 4: Figure S1.** Analysis workflow for analyzing CRC single-cell RNA-seq data. (A) Overview of two scRNA-seq datasets. ‘nFeature_RNA’ represents the number of genes measured in each cell, ‘nCount_RNA’ represents the sum of the expression of all genes measured in each cell, and ‘Percent.mt’ represents the percentage of mitochondrial genes measured. (B) Pearson’s correlation analysis of the sequencing depth and number of detected genes. (C) Principal component analysis (PCA) showed no significant outliers in all CRC samples. (D) The elbow plot shows that the curve tends to become smooth after 20 PCs. (E) After dimensionality reduction using the t-SNE algorithm, 33 cell clusters were identified. (F, G) Distribution plot and batch effect plot of all CRC samples. (H) The heatmap displays the top 10 marker genes of each cell cluster.

**Additional file 5: Figure S2.** Differences in immunotherapeutic-related factors among different ligand-receptor subtypes. (A-C) Violin plots presenting the expression of immune checkpoint molecules in the GSE39582, GSE17538 and TCGA-COREAD datasets. (D) A submap was used to match the clinical response to immune checkpoint blockade therapy in the bulk RNA-seq data. NR: no-response, R: response. *P < 0.05, **P < 0.01.

**Additional file 6: Figure S3.** Genomic alterations of the ligand-receptor subtypes. (A) Top 10 significantly mutated genes of three subtypes. (B) Proportion of mutations associated with targeted therapy for colorectal cancer in three subtypes. (C) Mutations in oncogenic signaling pathways. (D) Comparison of tumor mutation burden in subtypes. (E) The distribution of fraction genome altered. (F) Amplifications (red) or deletion (blue) regions and G-score of copy number variations (CNVs) in the chromosome of CRC patients. G-score that is proportional to the total magnitude of aberrations at each region. These significant amplifications and deletion regions were highlighted. (G) Enriched functions of CNVs related to gene expression in each subtype.

**Additional file 7: Figure S4.** Mutation background of CRC cell lines and the influence of pathway inhibitors. (A) CCK8 experiment to determine the effect of PI3K/AKT inhibitors (LY294002, PI-103), TNFA inhibitors (R-7050, Geraniin), TGF-Beta receptor inhibitors (A 83-01, SB-431542) and MAPK inhibitors(PD-98059, FR 180204)pathway on the viability of CRC cancer cells.(B) Pathways related mutation genes in the mutation profile of 4 CRC cell line.

**Additional file 8: Figure S5.** Bar plots of the interaction strength of ligand-receptor pairs associated with DC infiltration.

**Additional file 9: Figure S6.** Cell type identification in T cell and myeloid clusters. (A) t-SNE plots of 17183 T cells and 3848 myeloid cells. (B) The top 10 marker genes identifying myeloid cells are displayed in the heatmap. (C) Violin plot showing the expression of T cell marker genes. (D) t-SNE plots of 257 DCs identified three DC types. (E) The semisupervised trajectory of dendritic cells inferred by Monocle. (F) Violin plot showing the expression of dendritic cell marker genes. (G) The expression curve shows that the expression of the marker gene changes through the pseudotime expression mode.

**Additional file 10: Figure S7.** Selection of survival-related ligand-receptor pairs by LASSO regression and Cox regression. (A) The coefficient profile plot shows the screening process of parameters with increasing penalty factor values in the Lasso model. (B) The binomial deviance curve was plotted to select the optimal lambda value. The dotted vertical line on the left represents the best lambda value, and the line on the right represents the lambda value with standard error I. (C) The formula of the ligand-receptor risk score was constructed based on the multivariate Cox regression coefficients.

**Additional file 11: Figure S8.** Functional enrichment analysis of 30 survival-related ligand-receptor pairs. (A) GO-Biological Process, (B) GO-Cellular Component, (C) GO-molecular function, (D) Kyoto Encyclopedia of Genes and Genomes (KEGG).

## Data Availability

The data sets analyzed during the current study are available in the Gene Expression Omnibus (GEO, www.ncbi.nlm.nih.gov/geo/) and The Cancer Genome Atlas (TCGA) (https://portal.gdc.cancer.gov/).

## Abbreviations

LRI: Ligand-receptor interaction
CRC: Colorectal cancer
scRNA-seq: Single-cell RNA sequencing
IARC: International Agency for Research on Cancer
UMI: Unique molecular identifier
tSNE: T-distributed stochastic neighbor embedding
NPCs: Number of principal components
DC: Dendritic cell
DEP: Differentially expressed pair
KM: Kaplan–Meier
OS: Overall survival
TILs: Tumor-infiltrating lymphocytes
LASSO: Least absolute shrinkage and selection operator
