# Supplementary figures and images for: Delineation of colorectal cancer ligand-receptor interactions and their roles in the tumor microenvironment and prognosis
Source: J Transl Med. 2021 Dec 7;19:497. doi: 10.1186/s12967-021-03162-0 (PMC8650275; doi:10.1186/s12967-021-03162-0)

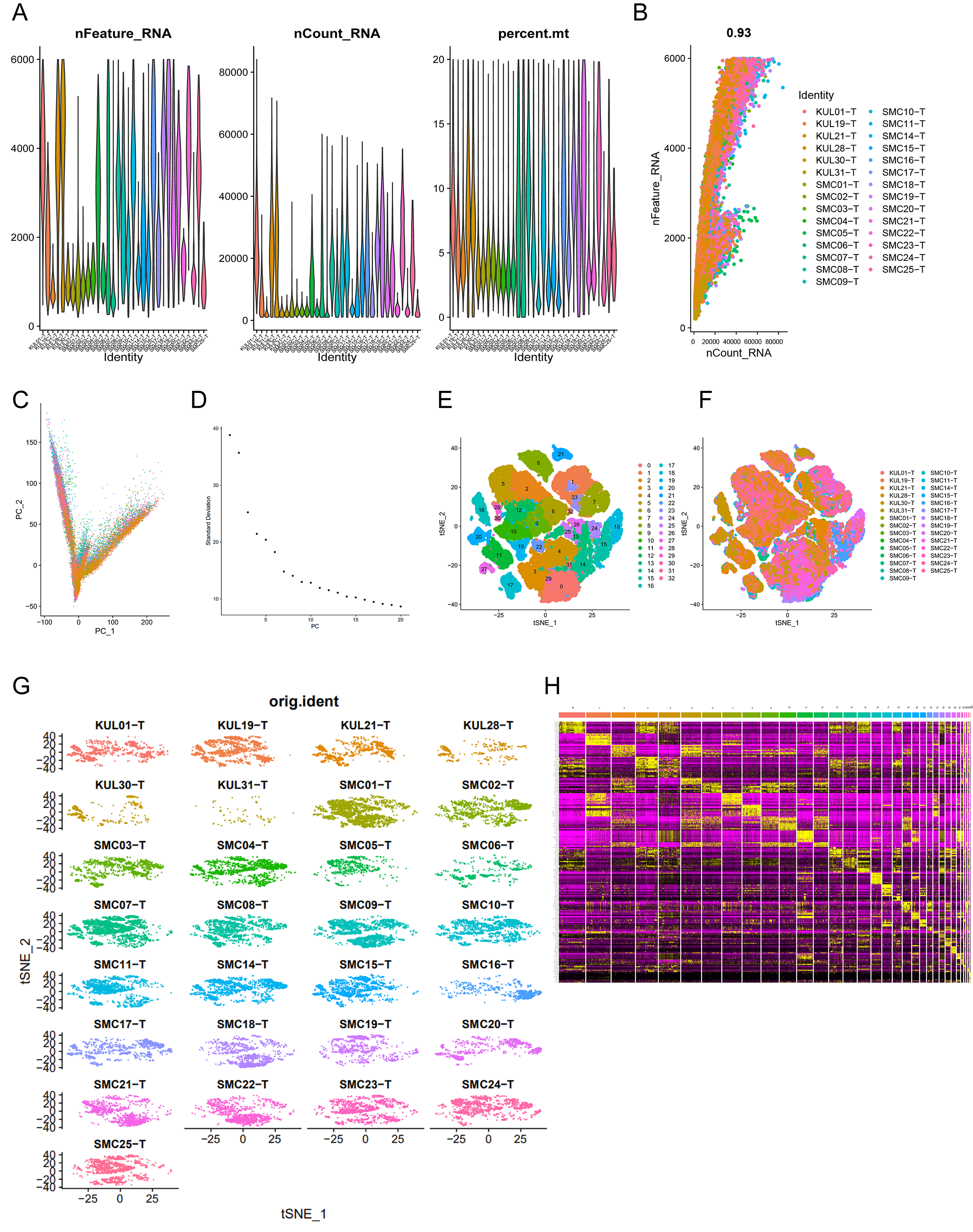

Supplement: Supplementary file 4 — Additional file 4: Figure S1. Analysis workflow for analyzing CRC single-cell RNA-seq data. (A) Overview of two scRNA-seq datasets. ‘nFeature_RNA’ represents the number of genes measured in each cell, ‘nCount_RNA’ represents the sum of the expression of all genes measured in each cell, and ‘Percent.mt’ represents the percentage of mitochondrial genes measured. (B) Pearson’s correlation analysis of the sequencing depth and number of detected genes. (C) Principal component analysis (PCA) showed no significant outliers in all CRC samples. (D) The elbow plot shows that the curve tends to become smooth after 20 PCs. (E) After dimensionality reduction using the t-SNE algorithm, 33 cell clusters were identified. (F, G) Distribution plot and batch effect plot of all CRC samples. (H) The heatmap displays the top 10 marker genes of each cell cluster. [file 12967_2021_3162_MOESM4_ESM.tif]

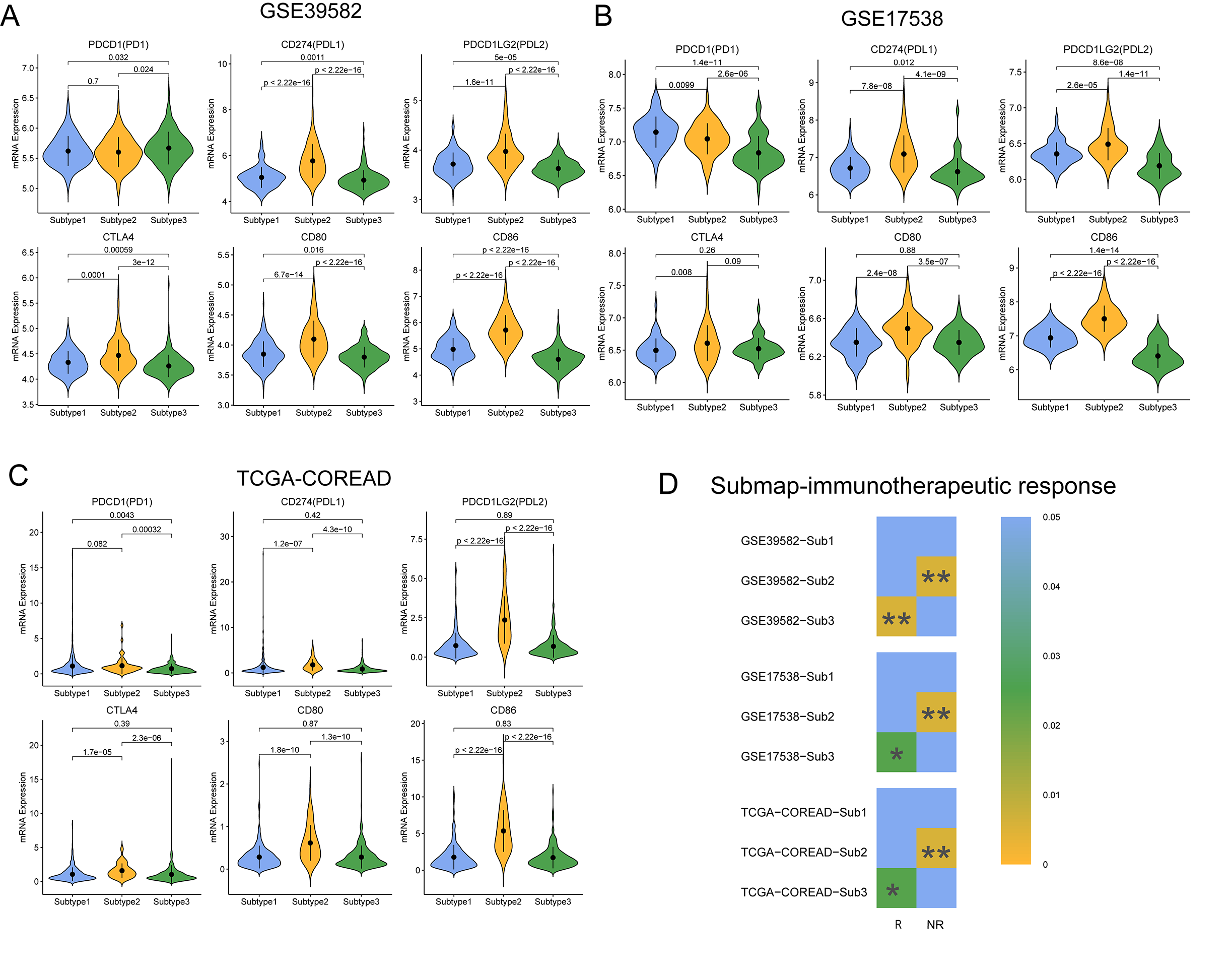

Supplement: Supplementary file 5 — Additional file 5: Figure S2. Differences in immunotherapeutic-related factors among different ligand-receptor subtypes. (A-C) Violin plots presenting the expression of immune checkpoint molecules in the GSE39582, GSE17538 and TCGA-COREAD datasets. (D) A submap was used to match the clinical response to immune checkpoint blockade therapy in the bulk RNA-seq data. NR: no-response, R: response. *P < 0.05, **P < 0.01. [file 12967_2021_3162_MOESM5_ESM.tif]

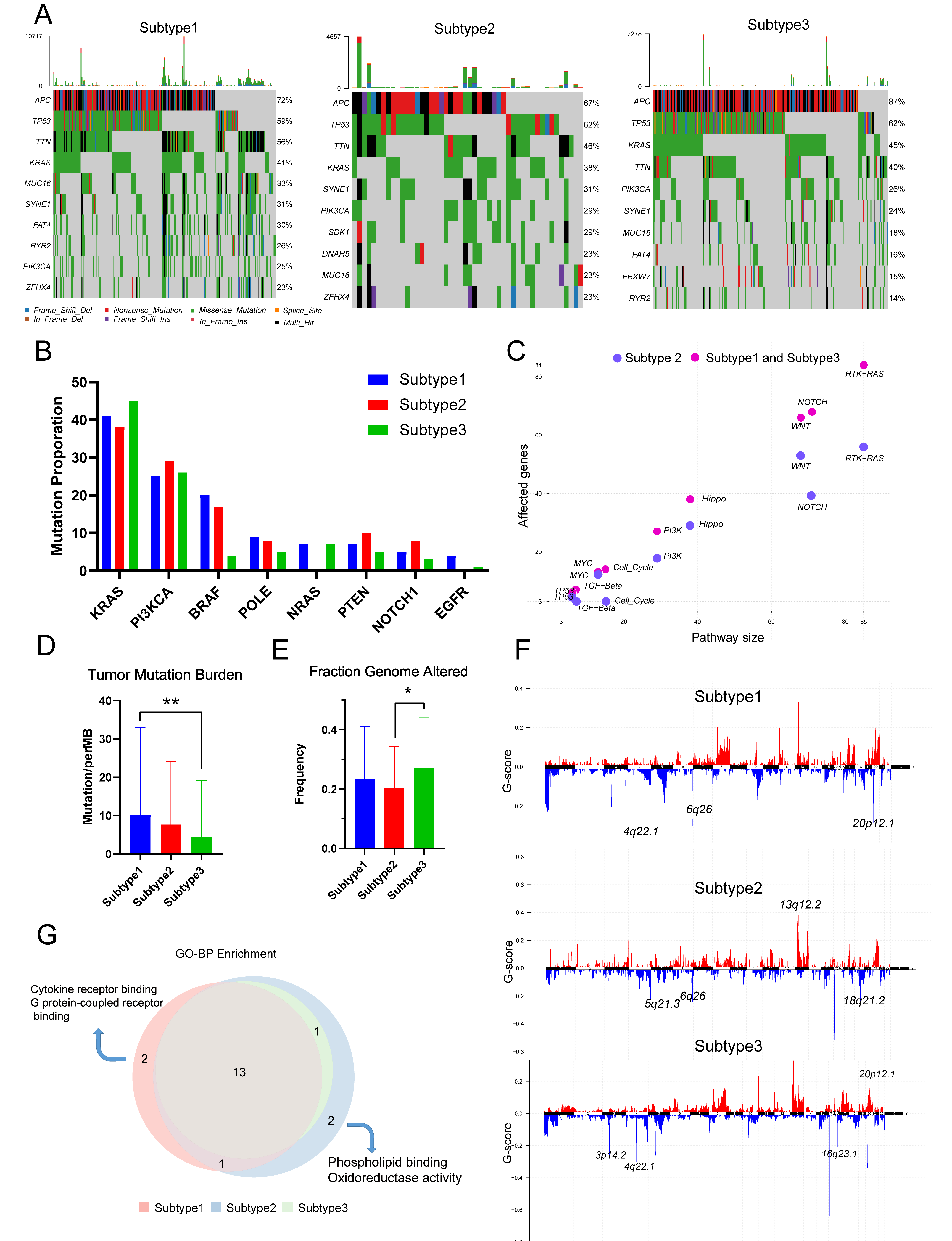

Supplement: Supplementary file 6 — Additional file 6: Figure S3. Genomic alterations of the ligand-receptor subtypes. (A) Top 10 significantly mutated genes of three subtypes. (B) Proportion of mutations associated with targeted therapy for colorectal cancer in three subtypes. (C) Mutations in oncogenic signaling pathways. (D) Comparison of tumor mutation burden in subtypes. (E) The distribution of fraction genome altered. (F) Amplifications (red) or deletion (blue) regions and G-score of copy number variations (CNVs) in the chromosome of CRC patients. G-score that is proportional to the total magnitude of aberrations at each region. These significant amplifications and deletion regions were highlighted. (G) Enriched functions of CNVs related to gene expression in each subtype. [file 12967_2021_3162_MOESM6_ESM.tif]

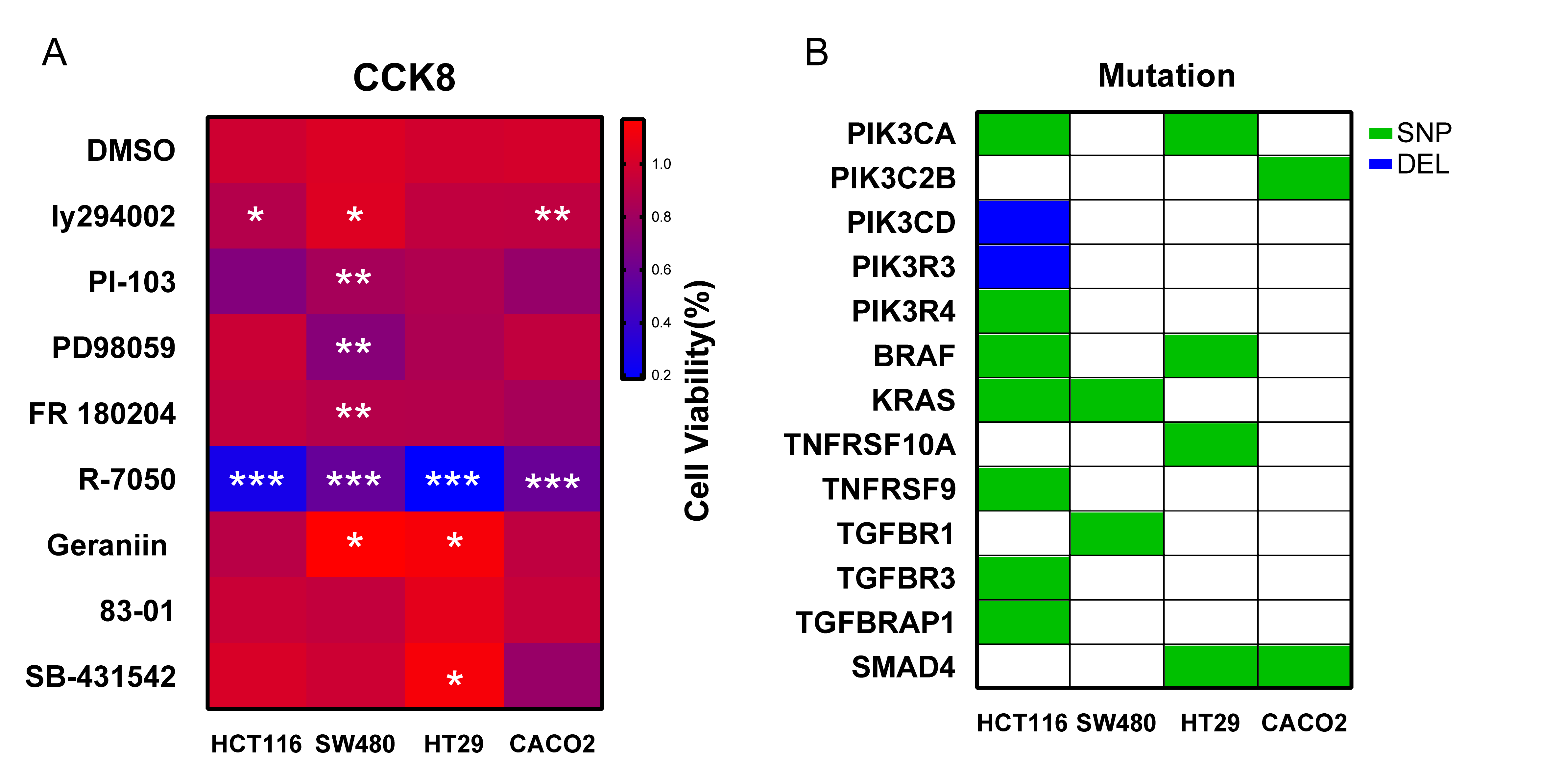

Supplement: Supplementary file 7 — Additional file 7: Figure S4. Mutation background of CRC cell lines and the influence of pathway inhibitors. (A) CCK8 experiment to determine the effect of PI3K/AKT inhibitors (LY294002, PI-103), TNFA inhibitors (R-7050, Geraniin), TGF-Beta receptor inhibitors (A 83-01, SB-431542) and MAPK inhibitors(PD-98059, FR 180204)pathway on the viability of CRC cancer cells.(B) Pathways related mutation genes in the mutation profile of 4 CRC cell line. [file 12967_2021_3162_MOESM7_ESM.tif]

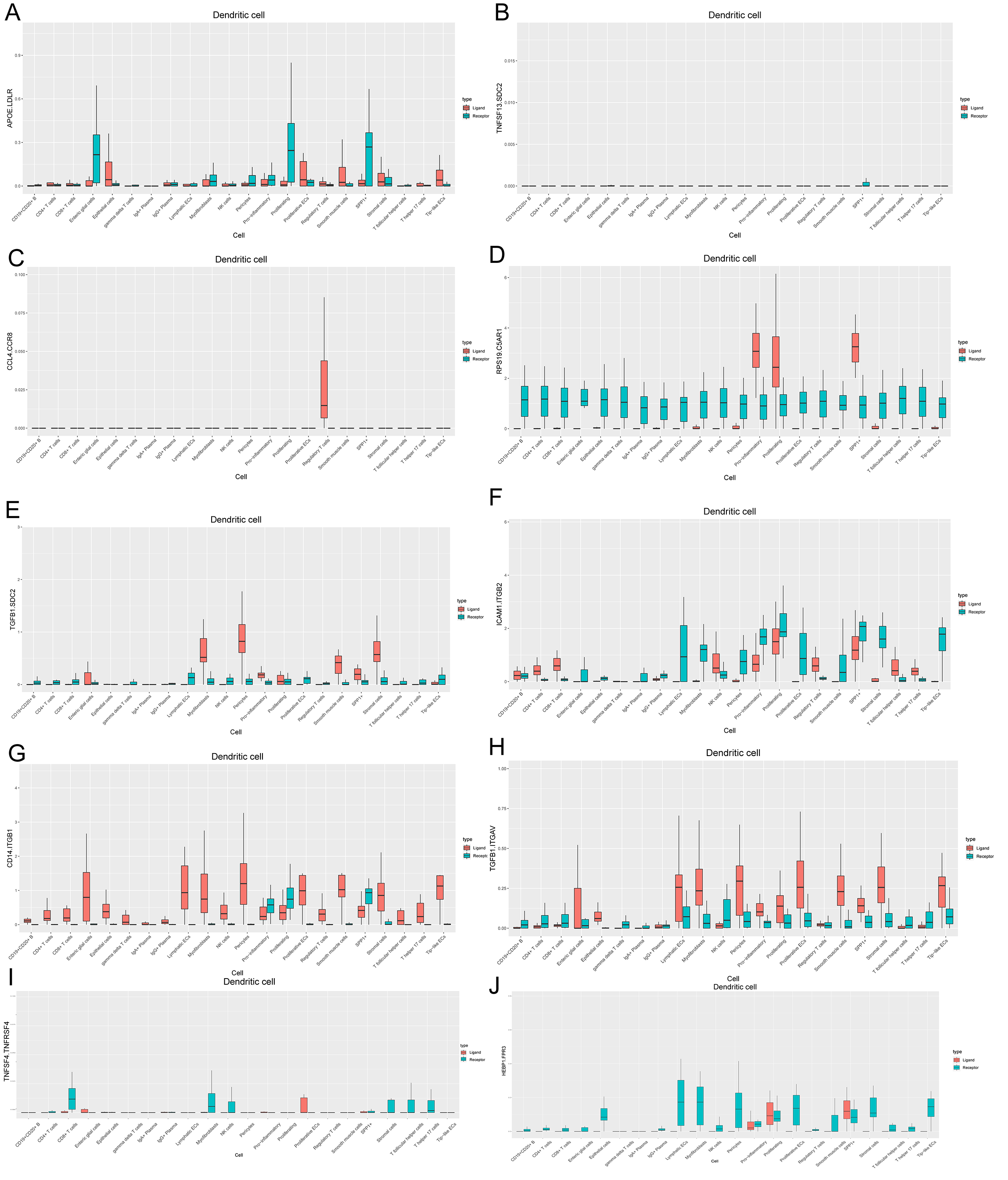

Supplement: Supplementary file 8 — Additional file 8: Figure S5. Bar plots of the interaction strength of ligand-receptor pairs associated with DC infiltration. [file 12967_2021_3162_MOESM8_ESM.tif]

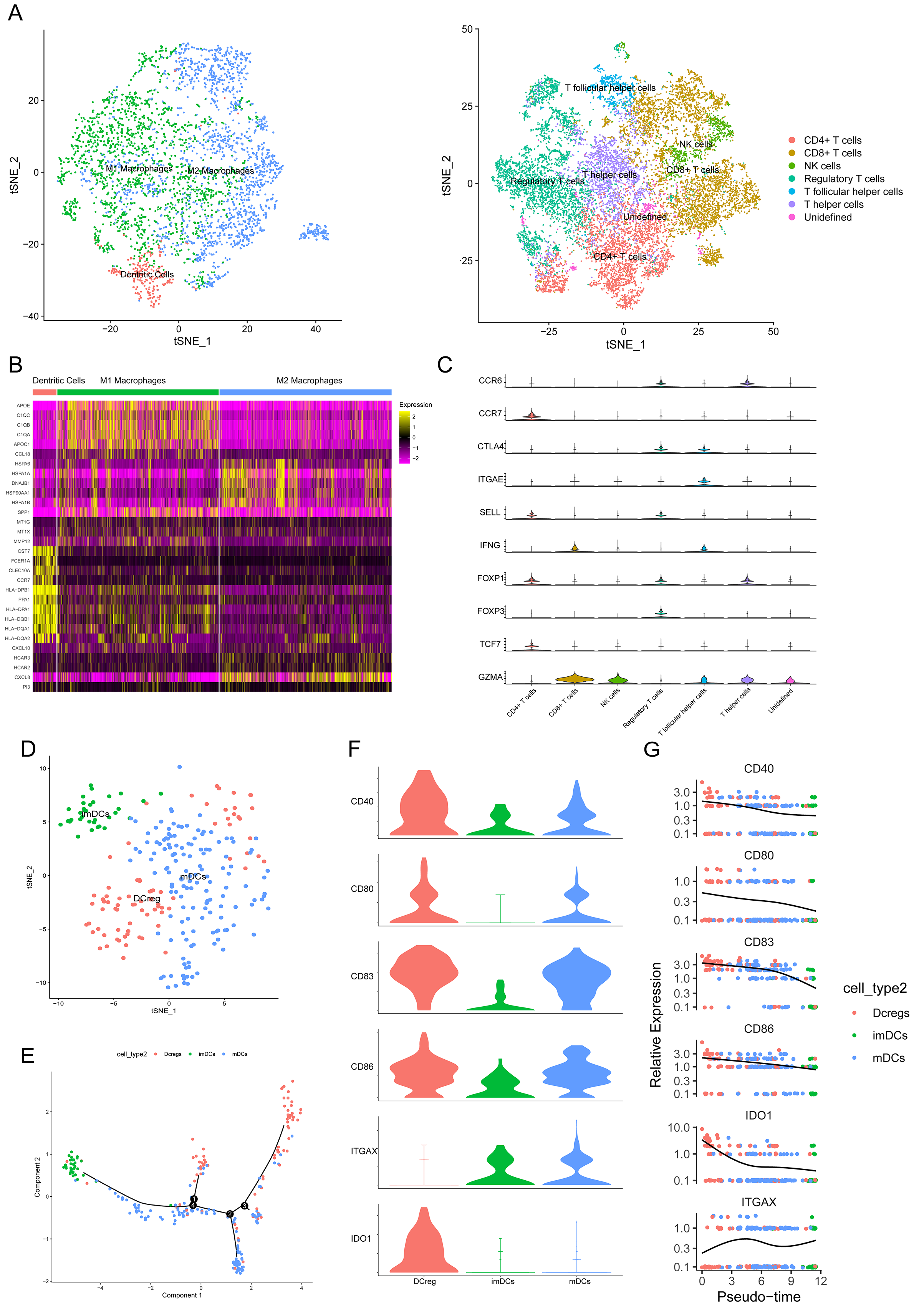

Supplement: Supplementary file 9 — Additional file 9: Figure S6. Cell type identification in T cell and myeloid clusters. (A) t-SNE plots of 17183 T cells and 3848 myeloid cells. (B) The top 10 marker genes identifying myeloid cells are displayed in the heatmap. (C) Violin plot showing the expression of T cell marker genes. (D) t-SNE plots of 257 DCs identified three DC types. (E) The semisupervised trajectory of dendritic cells inferred by Monocle. (F) Violin plot showing the expression of dendritic cell marker genes. (G) The expression curve shows that the expression of the marker gene changes through the pseudotime expression mode. [file 12967_2021_3162_MOESM9_ESM.tif]

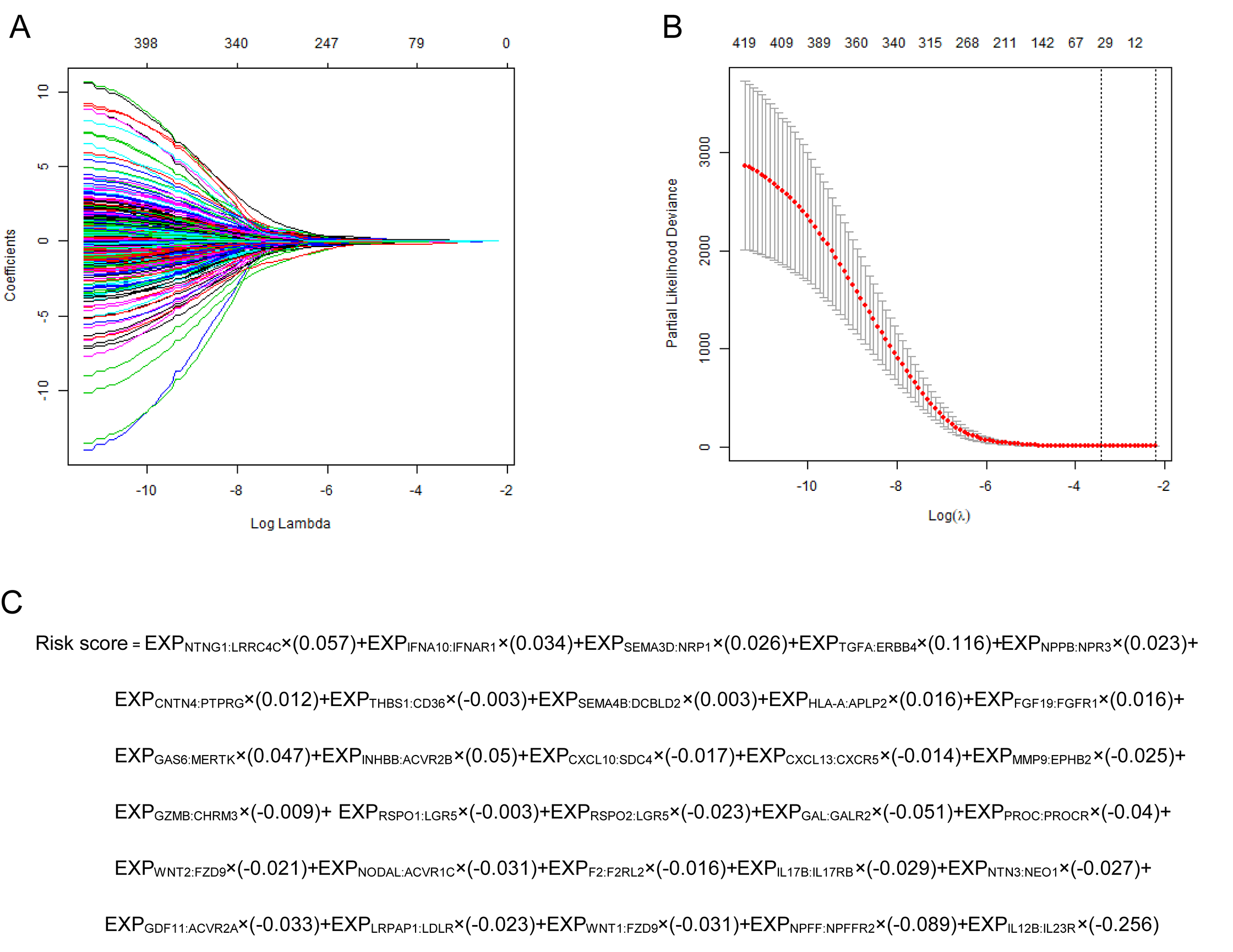

Supplement: Supplementary file 10 — Additional file 10: Figure S7. Selection of survival-related ligand-receptor pairs by LASSO regression and Cox regression. (A) The coefficient profile plot shows the screening process of parameters with increasing penalty factor values in the Lasso model. (B) The binomial deviance curve was plotted to select the optimal lambda value. The dotted vertical line on the left represents the best lambda value, and the line on the right represents the lambda value with standard error I. (C) The formula of the ligand-receptor risk score was constructed based on the multivariate Cox regression coefficients. [file 12967_2021_3162_MOESM10_ESM.tif]

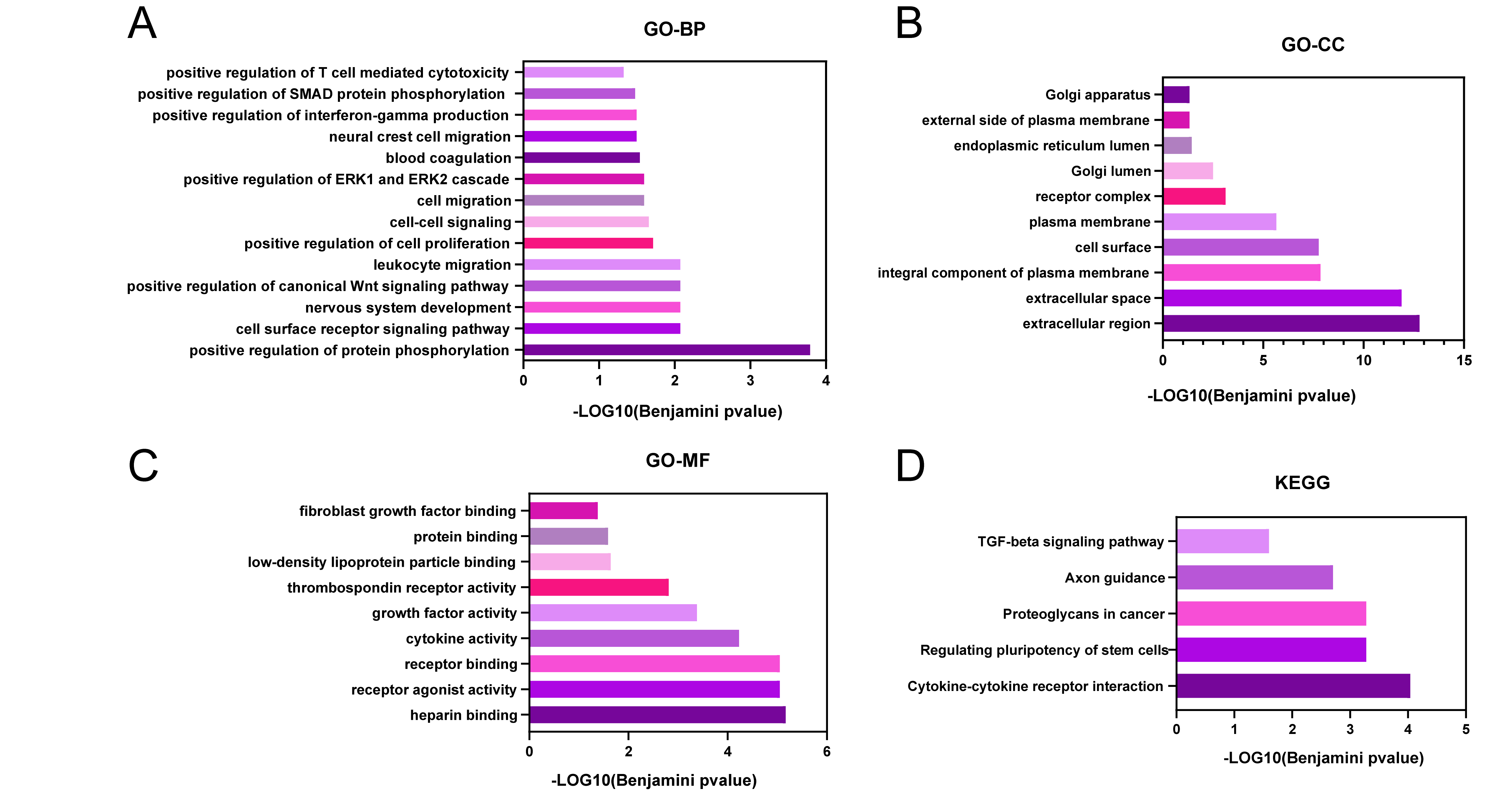

Supplement: Supplementary file 11 — Additional file 11: Figure S8. Functional enrichment analysis of 30 survival-related ligand-receptor pairs. (A) GO-Biological Process, (B) GO-Cellular Component, (C) GO-molecular function, (D) Kyoto Encyclopedia of Genes and Genomes (KEGG). [file 12967_2021_3162_MOESM11_ESM.tif]
